# Spatiotemporal Mapping of Interictal Spike Propagation: A Novel Methodology Applied to Pediatric Intracranial EEG Recordings

**DOI:** 10.3389/fneur.2016.00229

**Published:** 2016-12-19

**Authors:** Samuel B. Tomlinson, Camilo Bermudez, Chiara Conley, Merritt W. Brown, Brenda E. Porter, Eric D. Marsh

**Affiliations:** ^1^Department of Pediatrics, Division of Child Neurology, Children’s Hospital of Philadelphia, Philadelphia, PA, USA; ^2^School of Medicine and Dentistry, University of Rochester Medical Center, Rochester, NY, USA; ^3^Department of Neurology and Neurological Science, Stanford School of Medicine, Palo Alto, CA, USA; ^4^Department of Neurology, Perelman School of Medicine, University of Pennsylvania, Philadelphia, PA, USA

**Keywords:** epileptogenic zone, epilepsy surgery, interictal spike propagation, surgical outcome, pediatric epilepsy

## Abstract

Synchronized cortical activity is implicated in both normative cognitive functioning and many neurologic disorders. For epilepsy patients with intractable seizures, irregular synchronization within the epileptogenic zone (EZ) is believed to provide the network substrate through which seizures initiate and propagate. Mapping the EZ prior to epilepsy surgery is critical for detecting seizure networks in order to achieve postsurgical seizure control. However, automated techniques for characterizing epileptic networks have yet to gain traction in the clinical setting. Recent advances in signal processing and spike detection have made it possible to examine the spatiotemporal propagation of interictal spike discharges across the epileptic cortex. In this study, we present a novel methodology for detecting, extracting, and visualizing spike propagation and demonstrate its potential utility as a biomarker for the EZ. Eighteen presurgical intracranial EEG recordings were obtained from pediatric patients ultimately experiencing favorable (i.e., seizure-free, *n* = 9) or unfavorable (i.e., seizure-persistent, *n* = 9) surgical outcomes. Novel algorithms were applied to extract multichannel spike discharges and visualize their spatiotemporal propagation. Quantitative analysis of spike propagation was performed using trajectory clustering and spatial autocorrelation techniques. Comparison of interictal propagation patterns revealed an increase in trajectory organization (i.e., spatial autocorrelation) among Sz-Free patients compared with Sz-Persist patients. The pathophysiological basis and clinical implications of these findings are considered.

## Introduction

Interictal spikes are transient, high-amplitude discharges resolvable using both non-invasive (i.e., scalp) and invasive EEG electrodes. The presence of EEG spikes is strongly associated with epilepsy and contributes to the diagnosis; about 90% of epilepsy patients show EEG spiking compared to <1% of people without epilepsy (1). In the presurgical evaluation of intractable epilepsy patients, neurologists traditionally interpret spikes as focal biomarkers of pathological tissue that should be resected to ensure seizure freedom (2). However, evidence supporting this interpretation of spike activity is inconclusive (3). Resection of the spiking region has been associated with improved surgical outcomes (4, 5), though for many patients, persistent postoperative spiking is compatible with seizure freedom (6). Spikes are routinely observed in regions beyond the neurologist-defined seizure onset zone (SOZ), and the localization of spikes to the SOZ varies tremendously between patients and across time (7). Although spikes have been implicated in the formation of epileptic foci (8–11), several groups have suggested that periods of increased spikes actually protect against seizure occurrences (12, 13), challenging the assumption that “spikes beget seizures” (14).

Interictal spikes are sometimes recorded at multiple sites with discernible latency, suggesting that spikes can propagate rapidly across the cortex (15–18). In recent years, several groups have used spike propagation to visualize patterns of connectivity between remote neural populations (18, 19). This approach aligns with the emerging view of the epileptogenic zone (EZ) as a densely interconnected network capable of generating and sustaining seizures (20, 21). By mapping spike propagation through the cortex, several studies have correlated propagation trajectories with important clinical variables such as epileptic pathology (22) and SOZ localization (23–27). However, previous studies have been limited by important methodological concerns, e.g., the construction and overinterpretation of small, visually selected prototype spike datasets. Such methods require *a priori* information about spike events and have limited use in the surgical setting. While evidence is mounting for the clinical relevance of spike propagation, further study and methodological refinement are needed.

To assess the role of spike propagation in the presurgical evaluation, we developed a novel methodology for detecting, visualizing, and characterizing spike trajectories (or sequences) and applied it to a sample of 18 pediatric intracranial EEG recordings. After constructing large, unbiased spike datasets, we tested the hypothesis that the spatial organization of spike trajectories would reliably differ between patients with favorable (i.e., seizure-free) versus unfavorable (i.e., seizure-persistent) surgical outcomes. Additionally, we estimated the clinical impact of the methodology by comparing our approach to a more traditional spike analysis (i.e., mapping the focal density of spikes rather than their spatiotemporal spread). Our results suggest that patients with seizure-free outcomes exhibit more spatially organized interictal propagation patterns than patients with recurrent postoperative seizures. This work advances our understanding of propagating spike discharges and identifies a potential role for spike propagation as a quantitative surgical candidacy biomarker.

## Methods

### Patient Selection

The Children’s Hospital of Philadelphia (CHOP) Institutional Review Board approved this study. Each patient’s legal guardian signed written consent in accordance with the Declaration of Helsinki. Full-duration IEEG recordings were obtained from a database of Phase II presurgical evaluations performed at our institution between the years of 2002 and 2009. Each patient required intracranial EEG monitoring following unsatisfactory non-invasive localization of the epileptic foci. Of the 30 patients available for study, 18 patients (12 male, 6 female, mean age = 10.9 years, range = 3–20 years) met the inclusion criteria: (1) availability of detailed intraoperative photos; (2) unambiguous seizure markings; and (3) availability of ≥24 h of recordings. Patients were not screened for a particular clinical history, seizure semiology, seizure onset location, or electrographic onset pattern. All implants were clinically motivated without influence from this study. Retrospective review of patient charts provided detailed information regarding implantation site, pathology, etiology, and MRI description. Postsurgical outcomes were assessed by primary neurologists upon last patient contact (minimum post-operation = 2 years) and classified using Engel’s modified scale (28): Class 1 = seizure free; Class 2 = significant improvement; Class 3 = worthwhile improvement; and Class 4 = no improvement. Patients with complete postsurgical seizure freedom (Engel Score = 1, *n* = 9) were assigned to the “Sz-Free” group while patients experiencing persistent seizures (Engel Score ≥ 2, *n* = 9) were assigned to the “Sz-Persist” group.

### EEG Acquisition

Intracranial recordings were obtained using a Telefactor Beehive 32-128 channel CTE digital synchronized video-EEG system with 16-bit amplifiers and 200 Hz sampling rate (Astro Med Corp., West Warwick, RI, USA). Subdural grids and strips with embedded platinum electrodes (exposure diameter = 2.3 mm, interelectrode distance = 10 mm) were used for all patients (Adtech Medical Instrument Corporations, Racine, WI, USA). An outward-facing epidural electrode strip served as the online electrical reference. Data were subjected online to an analog antialiasing notch filter (60 Hz). De-identified intraoperative photos and diagrams provided by the surgical staff were used to construct 2D electrode maps for each patient. Cartesian coordinates were assigned to each channel using the interelectrode distance of 10 mm. Distances separating adjacent grids and strips were estimated using convenient anatomical landmarks. Electrode mapping was performed by an author (Samuel B. Tomlinson) blind to patient identity and confirmed by the senior author (Eric D. Marsh) prior to analysis. We acknowledge that this 2D approach does not capture the precise physical distance between electrode pairs, which is influenced by the 3D structure of the brain and the sulcal/gyral patterns beneath the arrays (29). However, approximating spatial relationships among channels using 2D schematics is common practice in the literature when coregistered MRI and CT imaging studies are unavailable.

### Seizure Annotation

Two experienced pediatric epileptologists (Brenda E. Porter and Eric D. Marsh) independently inspected each patient’s full IEEG records in referential montage and identified all seizures, marking the following parameters: (i) time of earliest electrical change (EEC) at ictal onset; (ii) time of unequivocal electrographic offset (UEO); and (iii) the seizure onset channels. To isolate interictal segments from full-duration recordings, seizure intervals (i.e., activity between EEC and UEO) were clipped and discarded, leaving only the non-seizure periods for analysis.

### Automated Spike Detection

In order to detect interictal spikes, we utilized an automated spike detector developed previously by our group and validated against human markings (30). The spike detection algorithm and the procedure for determining patient-specific thresholds are described thoroughly in the original publication. Briefly, spikes are identified based on stereotypic morphological features such as peak amplitude, spike duration, and the presence of a characteristic post-spike slow-wave (7). A previous validation study demonstrated that the spike detector performs comparably to expert epileptologists, with human verification of detected spikes exceeding an average of 75% (30). The detector is designed to process long EEG segments and output a two-column matrix encoding the channel index (column 1) and time (column 2) corresponding to each detected spike. For each patient, full interictal recordings were submitted to the spike detector. Spike detection was performed using MATLAB 2016a (Mathworks, Natick, MA, USA).

### Interictal Segmentation

Across patients, the duration of interictal EEG and the number of detected spikes were highly variable. In order to construct consistent spike datasets amenable to cross-subject comparisons, we divided each patient’s spike detector output into non-overlapping segments each containing 10,000 interictal spikes. Then, for each patient, 10 segments were randomly selected and concatenated to yield the final interictal spike dataset (spikes/patient = 100,000). Segments were chosen without regard for factors such as time-of-day, proximity to nearest seizure, and physiological state.

### Construction of Spike Frequency Maps

**Figure 1:**
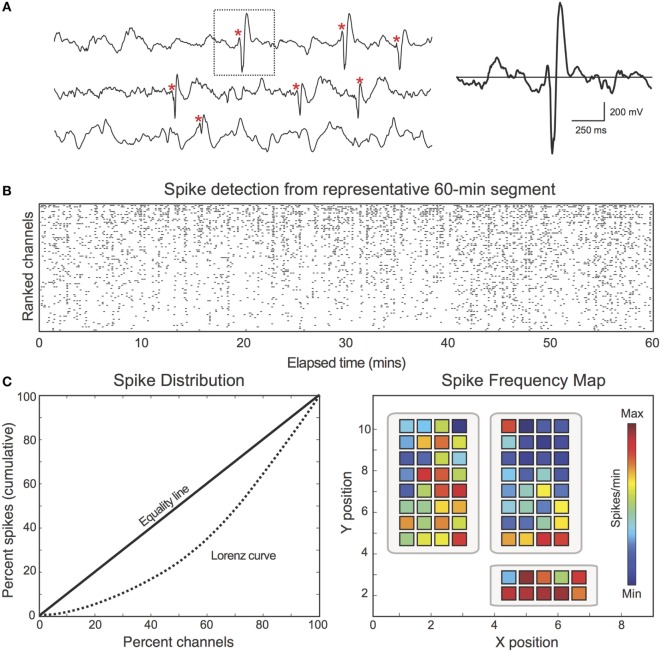
**Spike discharges were detected and encoded in spike frequency maps**. **(A)** For each patient, interictal spike discharges (red asterisk) were identified using an automated MATLAB detector. Spikes were identified based on characteristic morphological features and patient-specific thresholds. **(B)** Raster plot demonstrates the spike detector output from one representative 60-min EEG segment (Patient 18). Black dots correspond to the channel and time of each detected spike. The raster is sorted by spike count. **(C)** *Left*: spikes are unevenly distributed across channels. For illustrative purposes, the Lorenz curve (dotted) is used to describe the uniformity of the spike distribution. The Lorenz curve quantifies: “*X* percent of channels account for *Y* percent of the total spikes.” Deviation from the “Equality Line” (solid) confirms that the spike distribution is not uniform. *Right*: spike frequency maps were calculated by dividing each channel’s spike count by the total analyzed duration (spikes per minute).

First, we aimed to characterize the spatial distribution of spikes across the recording field (i.e., assessing how frequently spikes occurred at each electrode). Analyzing spikes in this manner closely parallels the traditional clinical interpretation of spike activity, with regions of high spike frequency typically marked as important areas for resection (2). For each patient, interictal spike frequency maps were generated by dividing each channel’s spike count by the total analyzed EEG duration (mins) (Figure 1). Frequency maps were coded with warmer colors representing higher spike frequency (spikes per minute) and cooler colors representing lower spike frequency (Figure 1C, right). In addition to spike frequency maps, spike distributions were represented using the Lorenz curve (31), which visualizes the distribution of assets (spikes) across the population (electrodes) (Figure 1C, left). The Lorenz curve was quantified using the Gini coefficient, which encodes the difference between the Lorenz curve and the linear “Equality Line” (i.e., a uniform distribution in which all channels exhibit the same percentage of the total spikes).

### Identifying Multichannel Spike Sequences

**Figure 2:**
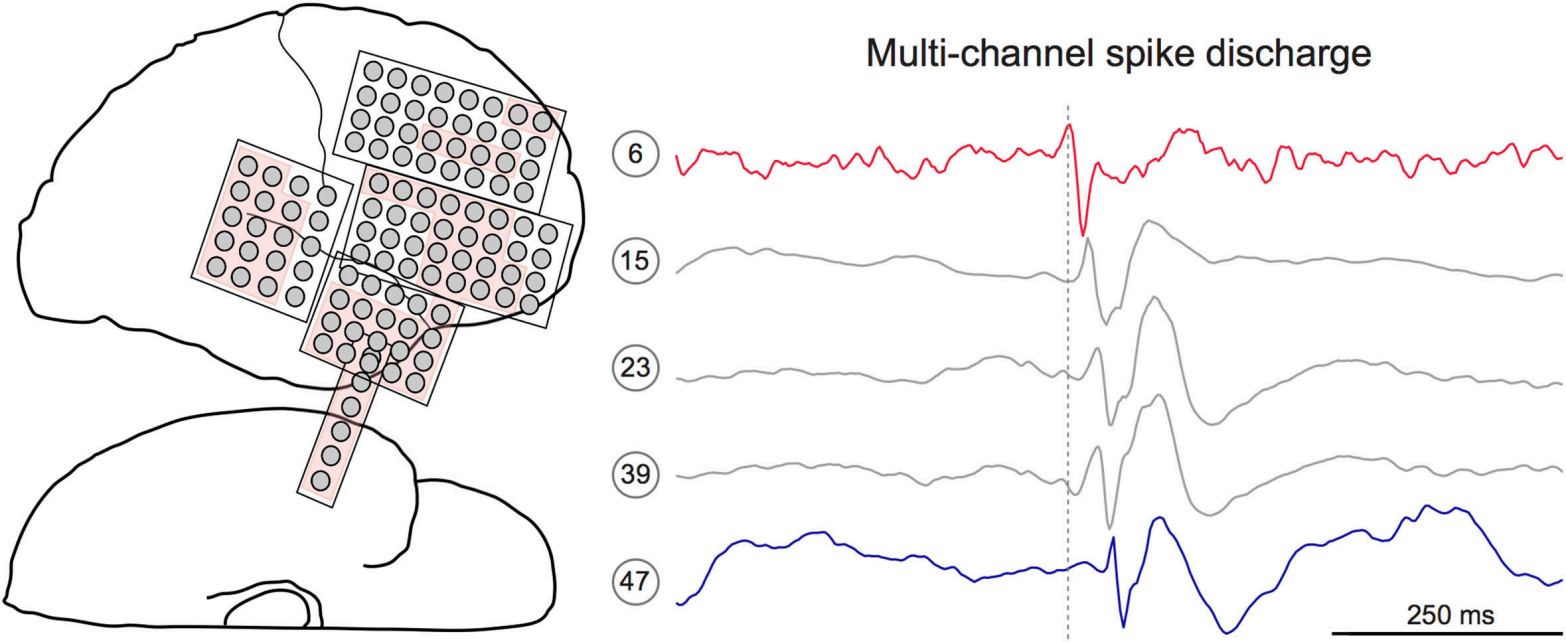
**Interictal spikes are sometimes observed at multiple electrode sites with discernible temporal latency**. *Left*: electrode schematic for Patient 12, a 7-year-old male with a temporal lobe implant. The neurologist-defined seizure onset zone is shown in red. *Right*: 1-s IEEG epoch containing a multichannel interictal spike discharge. Detailed inspection of discharge reveals subtle latency differences within the spike sequence. Here, the spike on channel 6 (red) precedes the spike on channel 47 (blue). Dotted gray line marks the peak time of the lead spike (red channel).

After visualizing the spatial density of mono-channel spike discharges, we next aimed to characterize the spatiotemporal propagation of spikes through the recording field. To do so, we developed a novel MATLAB algorithm that extracts multichannel sequences from the spike detector output (Figure 2). The algorithm initializes by defining the first detected spike as the “leader” of a candidate spike sequence (23). Successive spikes occurring within 50 ms of the leader were then appended to the candidate sequence. Fifty milliseconds was chosen based on previous studies showing that interictal spikes consistently propagate with 10–50 ms latencies (15, 23, 25, 32). Once a spike outside of 50 ms from the leader was encountered, this spike time was compared to the previous spike in the sequence. If the latency between the two events was ≤15 ms, the spike was added to the sequence. This additional parameter afforded the temporal flexibility needed to capture unexpectedly long spike sequences. A “terminating spike” was designated when the algorithm encountered a spike violating both temporal conditions (i.e., occurring ≥50 ms from leader and ≥15 ms from the previous spike). The terminating spike was then initialized as the leader of a new potential sequence, and the procedure restarted, continuing in this way until all spikes in the dataset were processed. To scrutinize the performance of the algorithm, two authors (Samuel B. Tomlinson and Eric D. Marsh) visually inspected the spike sequences extracted from random 60-min EEG segments for five patients. This preliminary review suggested that the majority of true-positive sequences included at least five mono-channel spikes while most false-positive detections included fewer than five spikes (data not shown). Therefore, only sequences containing ≥5 spikes were preserved for analysis.

### Applying Constraints to Spike Sequences

Two constraints were applied to the extracted spike sequences. First, due to the modest acquisition rate used in this study (200 Hz), spikes were sometimes detected with the same peak time. By default, the spike detector ordered these “tied” events according to increasing channel number, which does not reflect the actual path of the trajectory. To address this issue, tied spikes were rearranged based on spatial proximity (Euclidian distance) to the nearest non-tied event under the assumption that the probability of spike transmission decreases with distance. Second, propagation velocity is presumably limited by the neural conduction speed and the synaptic delay. It is therefore possible that spikes with minimal latency differences appearing at very remote sites belong to separate (but temporally overlapping) spike sequences. To account for this possibility, channels were grouped into partitions containing about four to eight neighboring electrodes. Each successive spike in a given sequence was required to occur within the same partition or a partition immediately adjacent to the previous spike. This procedure minimized the grouping of distinct spike sequences and limited the intrusion of unrelated but frequently spiking channels into the sequences. Because long-range neural connections are possible, a condition was included that allowed for connections traversing multiple partitions, if the connection was frequent. To identify frequent connections, we constructed a *channel*-by-*channel* matrix, *C*, such that *C*(*i*,*j*) encoded the number of times that a spike propagated from channel *i* to channel *j*. Then, when assessing whether the (*i*,*j*) connection was “frequent,” we divided *C*(*i*,*j*) by the sum of matrix row *i*. If this fraction exceeded 0.05 (i.e., when a spike is transmitted from channel *i*, it propagates directly to channel *j* at least 5% of the time), then the *C*(*i*,*j*) connection was deemed “frequent” and the spike was preserved. Partition assignments were made *via* inspection of de-identified intraoperative photos by Samuel B. Tomlinson and confirmed by Eric D. Marsh prior to the study.

### Eliminating Outlier Spike Trajectories

**Figure 3:**
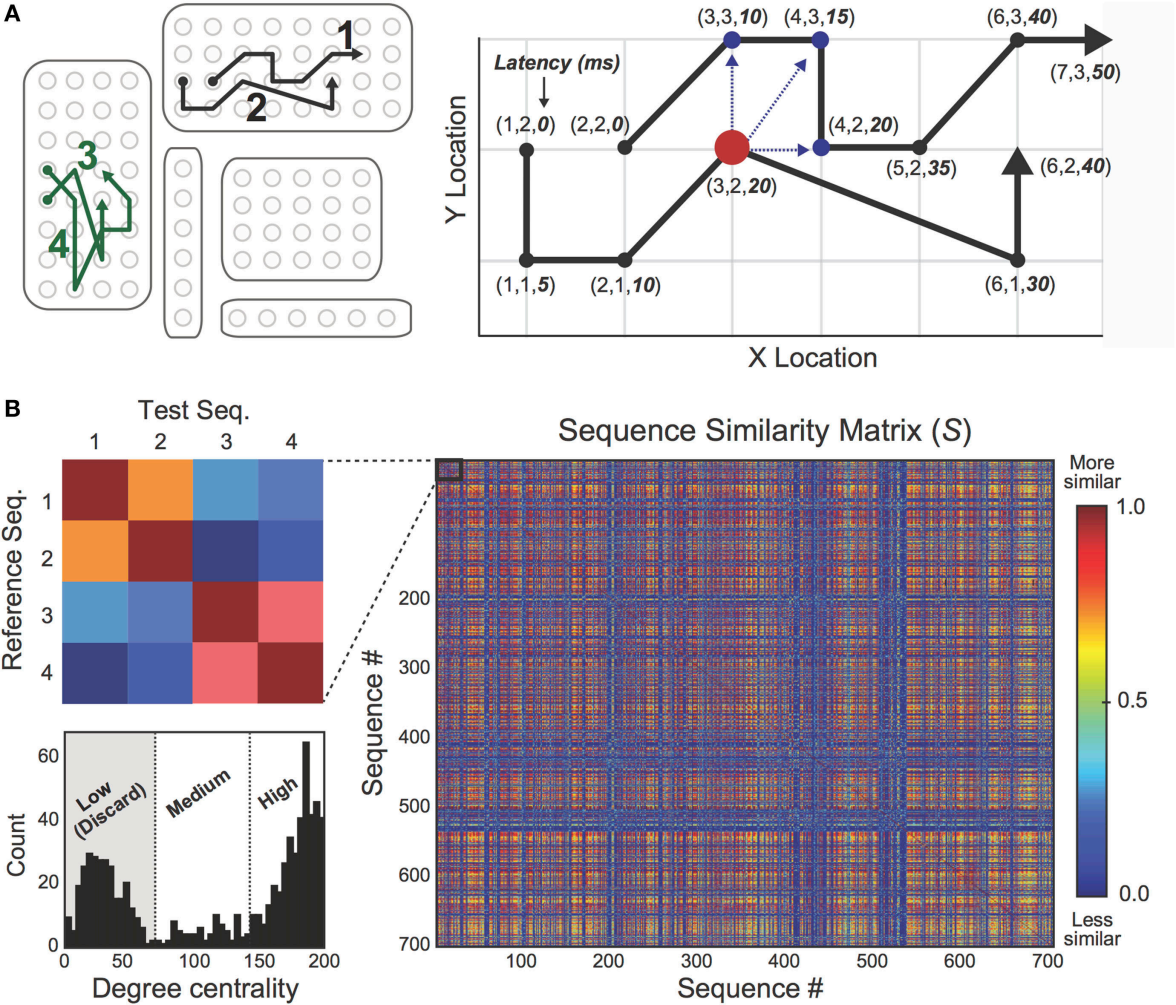
**Outlier spike sequences were eliminated using a sequence “cleaning” procedure**. **(A)** *Left*: four spike trajectories (labeled 1–4) are spatially mapped on the 2D electrode rendering. *Right*: a trajectory comparison algorithm was used to compare pairs of spike trajectories. Similarity scores were based on the extent of spatiotemporal overlap between trajectory pairs, ranging from min = 0 to max = 1. **(B)** Pairwise similarity scores were encoded in a *sequence*-by-*sequence* similarity matrix, *S*. The inset (left) shows the similarity mapping between trajectories 1 and 4 from part A. As expected, similarity scores between sequences 1 and 2 are high while sequences 3 and 4 share considerable spatiotemporal overlap. *Bottom left*: the degree distribution of matrix *S* was calculated in order to identify outlier sequences. A standard *k*-means clustering algorithm with fixed cluster count (*k*) of *k* = 3 was used to classify sequences as “Low Degree,” “Medium Degree,” and “High Degree.” Low Degree sequences shared minimal spatiotemporal overlap with the rest of the sequence set and were discarded as suspected outliers.

Identifying EEG spikes is an imperfect process, even for experienced human reviewers (7, 30). In this study, false-positive spike detections frequently occurred during periods of EEG artifact (e.g., during motor movements or ambient electrical noise), which were often falsely identified as propagating sequences. In order to discard erroneous sequence detections in an efficient and unbiased manner, we developed an unsupervised sequence “cleaning” procedure using a recent trajectory clustering algorithm (33). Briefly, the clustering algorithm computes a similarity score (min = 0, max = 1) between pairs of spike sequences based on their spatiotemporal overlap (Figure 3). For each patient, we identified all interictal spike sequences and submitted them to the comparison algorithm. Spike sequences were represented as a series of points, each taking the form (*x*-location, *y*-location, latency from lead spike). Then, a similarity score was computed between each pair of sequences in the set according to the following procedure. First, one sequence was designated as the “reference” sequence and the other was designated as the “test” sequence (Figure 3A, right). For each point in the reference sequence, the algorithm searched for data points (or “matches”) in the test sequence that satisfied predefined spatial and temporal thresholds (ss_thresh_ and tt_thresh_, respectively). Specifically, the algorithm found all points in the test sequence that (i) fell within 1.5 cm (Euclidean distance) from the reference point, and (ii) occurred within 15 ms of the reference point (i.e., occurred with similar latency from lead spike). Then, the “matched” point with the shortest Euclidean distance (*d*_match_) from the reference point was used to compute the following score: sim_point_ = 1 − *d*_match_/ss_thresh_, which ranged from min = 0 to max = 1. When no “matches” were found in the test sequence, sim_point_ = 0. This procedure was repeated for the remaining points in the reference sequence, and the average of the set of sim_point_ values constituted the similarity score between the reference and test sequences (again, min = 0 and max = 1). By iterating this process through all possible sequence pairs in the dataset, we constructed a non-symmetrical *sequence*-by-*sequence* similarity matrix, *S*, in which the *S*(*i*,*j*) cell contained the similarity score between sequences *i* and *j* (Figure 3B, right). Using this matrix, we computed the degree centrality (34) of each sequence to identify sequences sharing minimal spatiotemporal overlap with other discharges in the dataset. Based on the degree distribution (Figure 3B, bottom left), sequences were classified as “Low Degree,” “Mid Degree,” or “High Degree” using the MATLAB *k*-means clustering algorithm with a fixed cluster (*k*) count of *k* = 3, and all “Low Degree” sequences were eliminated as suspected outliers.

### Construction of Recruitment Latency Maps

**Figure 4:**
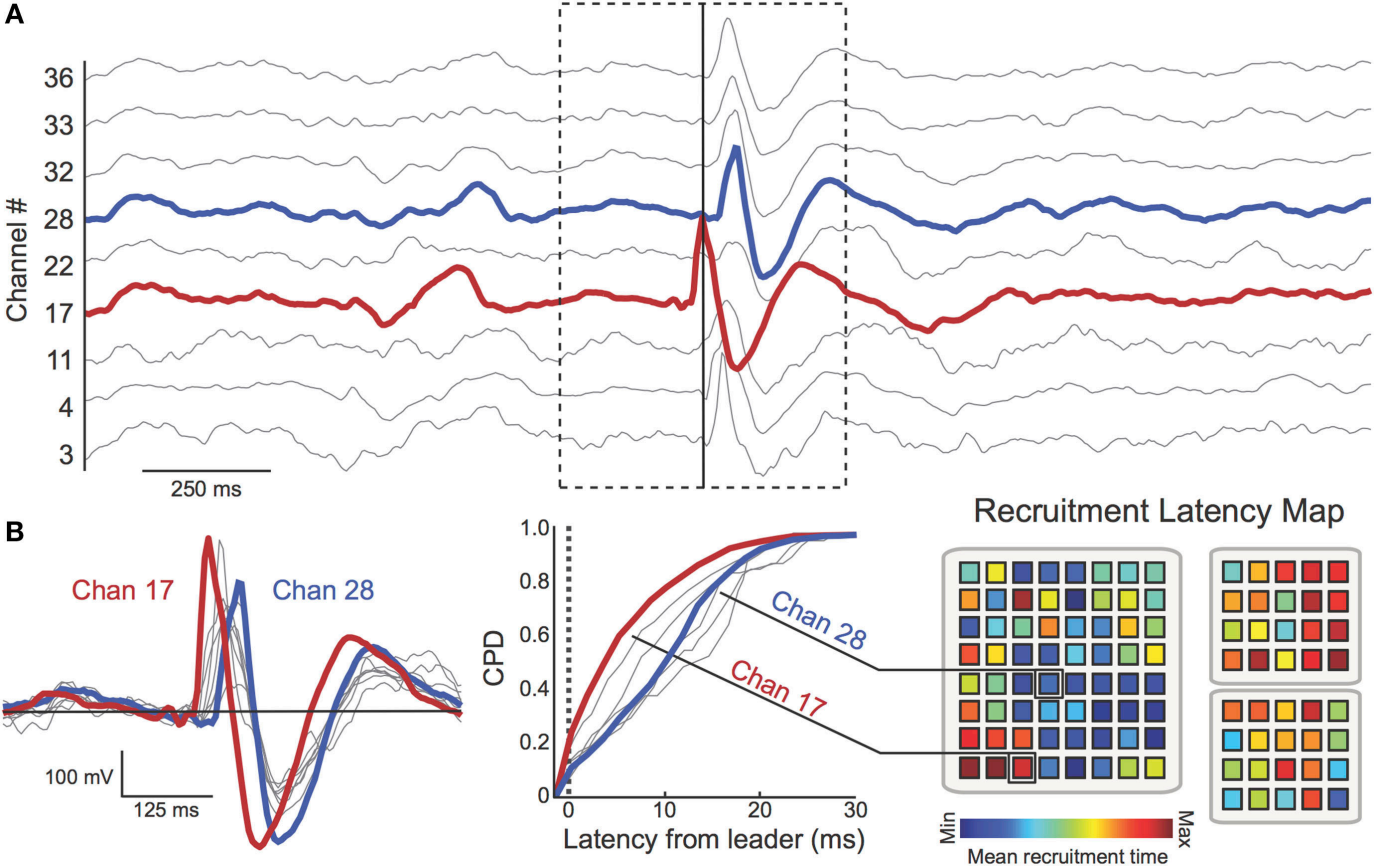
**Multichannel spike sequences were extracted using automated techniques**. **(A)** A 2.5 s epoch (Patient 17) containing a multichannel spike sequence (dotted box, nine channels shown). The time of the first peak in the sequence (red channel) is shown as a solid vertical line **(B)** *Left*: spike overlay (duration = 625 ms) reveals latency differences between spikes in the sequence. Here, channel 17 (red) is the “leader” (i.e., first spike) of the multichannel discharge. Channel 28 (blue) peaks considerably later in the sequence. *Middle*: cumulative probability distribution (CPD) encodes the tendency for each channel to occur at a given latency across all spike sequences. The heterogeneity of the CPD demonstrates the preference for channels to appear at different recruitment latencies. *Right*: the mean recruitment latency was computed for each channel (red = early recruitment, blue = late recruitment). Again, channel 17 tends to be recruited earlier in spike sequences than channel 28. Spatial organization of recruitment latency maps was characterized using the Moran Index. For this patient, channels appearing early in spike discharges (warm colors, “source” regions) cluster together while “sink” regions (cool colors) cluster together, resulting in a high Moran Index.

After extracting multichannel spike sequences, we aimed to characterize global patterns of spike propagation. To do so, we created “recruitment latency” maps for each patient (Figure 4). Recruitment latency maps were constructed by determining each electrode’s average temporal position (ms) within spike sequences (i.e., the average latency from the first spike in the discharge). This information was first represented as a cumulative probability distribution (CPD) encoding the tendency for each channel to occur at a given latency across all spike sequences (Figure 4B, middle). Channels that consistently appeared early in spike sequences thus exhibited a left-shifted CPD. Then, to visualize recruitment latencies across electrodes, recruitment latency maps were constructed (Figure 4B, right), with warmer colors representing shorter mean recruitment times (29). This approach allowed us to visualize the global movement of spikes from “source” regions with lower recruitment latencies (warmer colors) to “sink” regions with higher recruitment latencies (cooler colors).

### Assessing Spatial Organization of Spike Frequency and Recruitment Latency Maps

**Figure 5:**
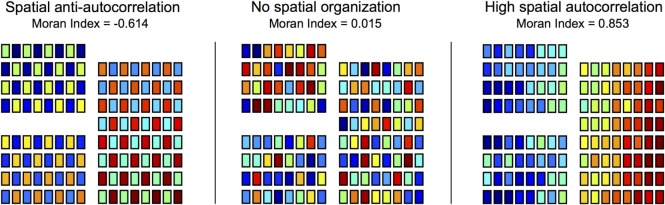
**Spatial organization of heat maps was quantified using the Moran Index**. Spike frequency maps and recruitment latency maps were characterized using the Moran Index, a spatial autocorrelation technique. Moran Indices ranged from −1 (perfect spatial anti-autocorrelation) to +1 (perfect spatial autocorrelation), with 0 corresponding to no patterns of spatial autocorrelation. To illustrate the Moran Index technique, simulated data are used to construct spatial maps (electrodes = 128) of varying degrees of spatial organization.

As described in the previous sections, two spatial maps were constructed per patient in order to describe interictal spike behavior: the spike frequency map (see Construction of Spike Frequency Maps), and the recruitment latency map (see Construction of Recruitment Latency Maps). Using these distinct approaches, we aimed to characterize the spatial organization of spike patterns. To examine the spatial organization of spike frequency maps, we asked: do regions of high (low) spike frequency cluster together in space? Similarly, for the recruitment latency maps, we assessed: do regions appearing early (late) in propagating discharges cluster together in space? Both questions could be readily examined using the Moran Index (35), a spatial autocorrelation measure that determines whether channels close in spatial proximity exhibit similar patterns of behavior (or in this case, similar spike frequencies/recruitment latencies). For the spike frequency maps, the Moran Index (*I*) was computed using the formula:

$$I=\frac{N}{\sum_{i}\sum_{j}w_{ij}}\frac{\sum_{i}\sum_{j}w_{ij}\left(F_{i}-F_{\mathrm{avg}}\right)\left(F_{j}-F_{\mathrm{avg}}\right)}{\sum_{i}{\left(F_{i}-F_{\mathrm{avg}}\right)}^{2}},$$

where *N* is the number of channels, *F_i_* is the spike frequency of channel *i* (spikes per minute), *F*_avg_ is the mean spike frequency across all channels, and *w_ij_* is the spatial “weight” between channels *i* and *j* (29). For recruitment latency maps, the same equation was used, but *F_i_* corresponded to the mean recruitment latency of channel *i* (ms) and *F*_avg_ was the mean recruitment latency across channels. In this study, if the Euclidean distance (*d*) between channels *i* and *j* (*d_ij_*) was ≤1.5 cm (i.e., the channels were immediately adjacent to one another, including diagonally), then *w_ij_* was set to 1/*d_ij_*. When *d_ij_* exceeded 1.5 cm, *w_ij_* was set to 0. An illustration of the Moran Index applied to various spatial maps is provided in Figure 5 using simulated data. As the simulation demonstrates the Moran Index ranges from −1 (perfect anti-autocorrelation between neighboring channels) to +1 (perfect autocorrelation between neighboring channels), with 0 indicating no pattern of spatial autocorrelation (29).

### Comparing Spike Frequency and Recruitment Latency Maps across Clinical Groups

Approaching spike activity from a spatial density perspective (i.e., spike frequency maps) versus a spatiotemporal trajectory perspective (i.e., recruitment latency maps) leads us to ask: does either approach provide insights into the likely surgical outcome of the patient? This question follows from our original hypothesis that spike propagation would reliably differentiate seizure-free and seizure-persistent patients whereas the more traditional spike frequency analysis would not capture such differences. To assess whether spike measures were related to surgical outcome, we conducted two comparisons between the study’s outcome groups (i.e., Sz-Free, *n* = 9; Sz-Persist, *n* = 9). In the first comparison, Moran Indices corresponding to spike frequency maps were compared across groups. In the second comparison, Moran Indices from the recruitment latency maps were compared across groups. Group comparisons were performed using the non-parametric Wilcoxon rank sum test with a Bonferroni-adjusted significance threshold of *p* = 0.05/2 = 0.025.

### Statistical Reporting

Unless otherwise noted, group-level statistics are reported as mean (μ) ± SD (σ), and *p*-values correspond to Wilcoxon rank sum tests.

## Results

The objective of this study was to assess the clinical utility of spike propagation as a biomarker for EZ organization and eventual seizure outcome in pediatric epilepsy surgery. To accomplish this goal, we developed a novel methodology for detecting, clustering, and visualizing global spike patterns from presurgical IEEG recordings. Then, we conducted a series of spike analyses on 18 patients (mean age = 10.9 ± 4.8 years) divided into two outcome groups: Sz-Free (Engel Score = 1, *n* = 9) and Sz-Persist (Engel Score, *n* = 9).

### Patient Characteristics

Clinical data from the patient cohort are presented in Table 1. Across patients, surgical implants ranged from 64 to 126 electrodes and did not differ in size between Sz-Free (102.4 ± 16.7 electrodes) and Sz-Persist (100.1 ± 16.2 electrodes) patients (*p* = 0.779). Implant locations were highly variable across patients and did not noticeably differ between groups. The percentage of channels included in the SOZ varied across patients (range = 4.0–100%) but did not differ between outcome groups (*p* = 0.436). Other variables including average follow-up duration, years since surgery, seizure frequency, and implant duration did not differentiate groups (data not shown).

**Table 1:** **Clinical characteristics of 18 study patients**.

Patient (*n* = 18)	Clinical details				Implant description				
	Age (years)	Gender	MRI	Engel	Implant	Electrodes	SOZ (%)	Analyzed minutes	Spikes (*n*)
Pt 01	9	M	LE	1	RF	126	6.35	1,051.92	50,000
Pt 02	18	M	NL	3	LF	100	4.00	338.28	100,000
Pt 03	7	M	NL	4	RF	99	41.41	153.56	100,000
Pt 04	16	F	NL	4	RF	94	11.70	445.82	100,000
Pt 05	20	F	LE	1	LF, LT	99	22.22	198.65	100,000
Pt 06	10	M	NL	3	LF	108	29.63	681.29	100,000
Pt 07	11	M	LE	3	LF	124	20.97	446.09	100,000
Pt 08	15	F	LE	3	RF	64	25.00	455.23	50,000
Pt 09	11	F	NL	1	RF	116	15.52	189.38	100,000
Pt 10	5	M	LE	1	LF	92	46.67	396.90	100,000
Pt 11	12	M	LE	1	L-Hemi	108	26.85	86.65	100,000
Pt 12	7	M	NL	4	RT	110	59.09	422.02	100,000
Pt 13	8	M	LE	4	RF	98	100.00	1,028.38	100,000
Pt 14	6	F	LE	4	RF	104	49.04	797.29	100,000
Pt 15	16	M	NL	1	RF	116	9.48	2,205.93	100,000
Pt 16	3	F	LE	1	RT	74	45.95	1,494.38	100,000
Pt 17	8	M	NL	1	LT	107	27.10	916.07	100,000
Pt 18	14	M	LE	1	RF	84	28.57	700.18	100,000
Sz-Free	10.89 ± 5.35	–	–	–	–	102.4 ± 16.7	25.4 ± 14.2	804.5 ± 702.8	–
Sz-Persist	10.89 ± 4.43	–	–	–	–	100.1 ± 16.2	37.9 ± 29.2	529.8 ± 262.8	–
*p*-value	0.947	–	–	–	–	0.779	0.436	0.667	–

*Age (years) at time of operation ranged from 3 to 20 years (M = 10.89, SD = 4.76 years). Patients with complete postsurgical seizure freedom (Engel Score = 1) were designated as “Sz-Free” (*n* = 9) while patients exhibiting recurrent seizures (Engel Score ≥2) were assigned to the “Sz-Persist” group (*n* = 9). *p* values for group comparisons were computed using Wilcoxon rank sum tests. L, left; R, right; F, frontal; T, temporal; Hemi, hemisphere; SOZ, seizure onset zone; LE, lesional; NL, non-lesional*.

### EEG Extraction and Spike Detection

Seizures were clipped from full-duration EEG datasets, leaving only the non-seizure windows for analysis (Table 2). For each patient, 10 segments of 10,000 interictal spikes were randomly extracted from the interictal spike detector output. Across patients, the duration of EEG (minutes) encompassed in the spike dataset varied dramatically but did not differ between Sz-Free (804.5 ± 702.8 min) and Sz-Persist (529.8 ± 262.8 min) groups (*p* = 0.667). Note from Table 1 that two patients (Patient 01, Sz-Free; Patient 08, Sz-Persist) had only five available interictal segments, yielding 50,000 spikes for analysis. Reanalyzing the data using five segments for each patient did not appreciably change the results presented below (Figure S1 in Supplementary Material).

**Table 2:** **The spatial density (“spike frequency analysis”) and spatiotemporal propagation (“recruitment latency analysis”) of interictal spike discharges were examined**.

Patient (*n* = 18)	Spike frequency analysis		Recruitment latency analysis		
	Spike density (spikes/chan/min)	Moran Index	Total sequences (*n*)	Sequence frequency (seqs/min)	Moran Index
Pt 01	0.377	0.527	135	0.1283	0.501
Pt 02	2.956	0.403	2,224	6.5745	0.307
Pt 03	6.578	0.284	739	4.8125	0.282
Pt 04	2.386	0.589	774	1.7361	0.338
Pt 05	5.085	0.427	1,018	5.1247	0.411
Pt 06	1.359	0.493	806	1.1831	0.349
Pt 07	1.808	0.153	993	2.2260	0.182
Pt 08	1.716	0.417	1,076	2.3636	0.178
Pt 09	4.552	0.488	1,845	9.7421	0.451
Pt 10	2.739	0.304	1,680	4.2328	0.832
Pt 11	10.685	0.705	2,292	26.4504	0.368
Pt 12	2.154	0.300	2,652	6.2840	0.411
Pt 13	0.992	0.335	916	0.8907	0.272
Pt 14	1.206	0.525	1,072	1.3445	0.151
Pt 15	0.391	0.203	1,323	0.5997	0.425
Pt 16	0.904	0.465	1,895	1.2681	0.437
Pt 17	1.020	0.218	923	1.0076	0.316
Pt 18	1.700	0.324	1,240	1.7710	0.283
Sz-Free	3.05 ± 3.35	0.407 ± 0.161	1,372.3 ± 643.8	5.59 ± 8.39	0.447 ± 0.160
Sz-Persist	2.35 ± 1.70	0.389 ± 0.136	1,250.2 ± 692.7	3.05 ± 2.23	0.275 ± 0.088
*p*-Value	0.730	0.863	0.340	0.863	0.003

*Patient-wise results from the two spike analyses are displayed. Patients exhibited highly variable spike density (spikes per channel per minute) and sequence frequency (sequences per minute), though differences between groups were not observed. Sz-Free patients exhibit significantly more organized recruitment latency maps (Moran Index) than Sz-Persist patients (Wilcoxon rank sum test, *p* = 0.003)*.

### Spatial Organization of Spike Frequency Maps Does Not Differ by Surgical Outcome

**Figure 6:**
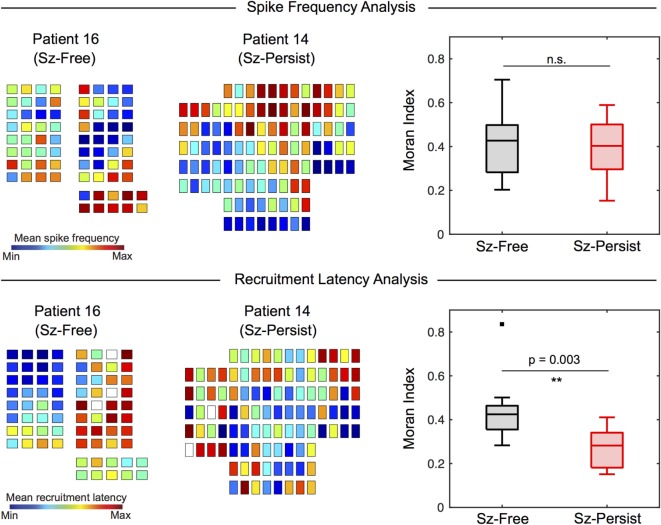
**Recruitment latency maps (but not spike frequency maps) differentiate Sz-Free and Sz-Persist groups**. The spatial organization of spike frequency maps and recruitment latency maps were assessed using the Moran Index. Box plots are used to display group results (box = 25th–75th percentile, horizontal line = median, whiskers = 5th–95th percentile). The spatial organization of spike frequency maps (*top*) does not differentiate clinical groups (Wilcoxon rank sum test, *p* = 0.863). When recruitment latency maps were examined (lower), we observed significantly increased spatial organization (Moran Index) among Sz-Free (0.447 ± 0.160) compared with Sz-Persist (0.275 ± 0.088) patients (Wilcoxon rank sum test, **p = 0.003). n.s., not significant.

Interictal spike frequency maps (spikes per minute) were constructed for each patient to visualize the distribution of spike events across channels. The spatial organization of spike frequency maps (quantified by the Moran Index) varied substantially across patients (Table 2). When surgical outcome groups were compared (Figure 6, top), Moran Indices (min = −1, max = 1) were similar for Sz-Free (0.407 ± 0.161) and Sz-Persist (0.389 ± 0.136) groups (*p* = 0.863). This result suggests that the spatial organization of spike frequency maps was not associated with surgical outcome.

### Spatial Organization of Recruitment Latency Maps Is Increased Among Patients with Favorable Surgical Outcome

We applied our novel spike propagation trajectory methods to the interictal detector output. Consistent with previous studies (17, 18), the frequency of multichannel spike sequences (sequences per minute) varied tremendously across patients (Table 2). The average sequence frequency across patients was 4.32 ± 6.10 sequences/min, with no differences between Sz-Free (5.59 ± 8.39 sequences/min) and Sz-Persist (3.05 ± 2.23 sequences/min) groups (*p* = 0.863). To characterize the global flow of propagating spikes across the recording field, recruitment latency maps were constructed by determining each channel’s average temporal position within spike sequences. When recruitment latency maps were compared across outcome groups (Figure 6, bottom), we observed significantly increased spatial organization (Moran Index) among Sz-Free (0.447 ± 0.160) compared with Sz-Persist (0.275 ± 0.088) patients (*p* = 0.003).

## Discussion

Defining and deciphering the network structure of the EZ is critical for improving the surgical management of intractable seizures. In this study, we examined connectivity patterns within the epileptic brain by mapping the propagation of intracranially recorded spike discharges in pediatric epilepsy surgery patients. Using a spatial autocorrelation approach, we found that the global organization of spike trajectories reliably distinguished patients with favorable versus unfavorable surgical outcomes. Further, we found that group differences were not observed using a more traditional (i.e., density-based) approach to spike activity, suggesting that our technique elucidates group differences that are inaccessible to spatial density analyses.

### Methodological Advancements

The novel methodology introduced here immediately advances the study of interictal spike propagation in several important ways. First, our method is the only published study that combines a logical automated spike propagation algorithm with a sophisticated trajectory “cleaning” procedure, allowing us to examine spike trajectories across the entire interictal recording without introducing overwhelming artifact to the dataset. In stark contrast, most previous studies have limited their analyses to brief, manually selected EEG segments with prototypic spike activity, which prohibits analysis of spike patterns across the full recording period and leads to sampling biases. Indeed, recent work finds that spikes originating from the same lead region follow highly variable trajectories (22), suggesting that limited samples of spikes may provide misleading information about the overall propagation patterns. Next, this study is among the first to examine the relationship between presurgical spike propagation and postsurgical seizure outcome. Demonstrating that spike propagation analyses can disentangle surgical outcome groups is an important step for translating this paradigm to the clinical setting. Additionally, although several groups have developed source reconstruction models of propagating spikes in EEG (17, 25, 27, 32, 36) and MEG recordings (37–39), few have attempted to capture the state of the entire recording field with a single global metric. By applying the Moran Index to the recruitment latency maps constructed for each patient, we reduced the dimensionality of the multivariate electrode grid to a global statistic with an intuitive interpretation (i.e., spatial organization). This approach is advantageous compared to more complex analyses because it may allow clinicians to efficiently incorporate spike propagation into the workflow of the presurgical evaluation. Finally, our study is unique in demonstrating the superiority of the spike propagation approach over a more traditional spike analysis based on spatial density. While the methodology of this study immediately advances the field in these regards, the clinical efficacy of our technique must be validated in a larger study with prospectively collected IEEG data from both adult and pediatric patients.

### Spike Propagation Is More Spatially Organized in Patients with Favorable Outcomes

In our pediatric cohort, patients with seizure-free outcomes exhibited increased spatial organization of spike trajectories relative to patients with persistent seizures. This finding aligns with recent work by Martinet et al. (29), who mapped the spatiotemporal propagation of neocortical seizures and demonstrated that more consistent and organized ictal recruitment patterns were associated with improved surgical outcomes. Critically, these authors relied on a similar application of the Moran Index to analyze ictal recruitment latency maps, maximizing the comparability of the findings. The concordance of these studies is somewhat surprising given that the relationship between spikes, seizures, and surgical outcome is highly inconsistent across studies (10). However, researchers have yet to determine whether spike propagation and seizure propagation networks overlap in space, which may reconcile the findings of these two studies. Critically, the analysis of spike propagation has practical benefits over seizure propagation, as interictal spikes are more readily acquired and may reduce the necessary implant time in patients undergoing Phase II surgical evaluation. Nonetheless, the findings bolster one another in suggesting that epileptiform propagation elucidates patterns of EZ connectivity that may offer insights into the eventual surgical outcome of the patient.

### Spike Propagation Reveals Group Differences That Are Not Observed in the More Traditional, Density-Based Spike Analysis

We propose that spike propagation analyses differ from more traditional spike density analyses because the former captures the network structure and organization of the EZ in a manner inaccessible to the latter. Indeed, in recent years, network analysis of EEG recordings has emerged as a major area of research (40, 41). Researchers have used principles from network science and graph theory to localize the EZ (42, 43), examine the events surrounding ictal onset (44) and predict surgical outcome (45) with increasing levels of success. Spike propagation is an intuitive method for visualizing connectivity patterns within the EZ, allowing researchers to quantify both the strength and directionality of pairwise connections (i.e., how often is a spike transmitted from channel *A* to *B*, and vice versa). The results from this study suggest that spike propagation is a valuable and potentially under-utilized tool for assessing the network configuration of the EZ.

### Future Directions and Limitations

This study provides evidence for the relevance of spike propagation in the surgical management of epilepsy patients. Although the methodological and clinical advances of this study are significant, future steps must be taken to bolster our results. First, a major limitation of this study is the small number of patients included in the analyses. While small samples are common in both adult and pediatric IEEG studies, validating the clinical findings of our study requires a prospective study with considerably larger group sizes. Second, the reliance on two-dimensional spatial electrode maps is a potential limitation of the design. While recent procedures have been introduced to localize electrodes using coregistered presurgical CT imaging, many IEEG studies continue to rely on 2D electrode schematics. We posit that representing the electrode array in two dimensions is a reasonable simplification, given our interest in mapping spike propagation across the cortical surface. However, future studies using stereo-EEG electrodes or synthetic depth electrodes (MEG) may reveal important contributions from deep neural sources, and validating electrode positions using co-registration technologies may strengthen the present results. Finally, as with all IEEG studies, the study is limited by the clinically chosen electrode locations and may not sample the full extent of cortical spiking.

### Conclusion

This study presents a novel methodology for detecting, extracting, and characterizing presurgical spike propagation patterns. Our results demonstrate the potential clinical utility of spike propagation in assessing surgical candidacy and characterizing the EZ in pediatric epilepsy surgery. The findings of this study represent a positive step toward a presurgical biomarker of seizure freedom and highlight the need for improved understanding of interictal spike activity in the clinical work-up for epilepsy surgery. This work justifies a large-scale prospective study that will further elucidate the role of spike propagation in the surgical management of refractory neocortical seizures.

## Author Contributions

The following contributions were made by the authors: experimental hypothesis and study design (ST, EM, and BP), data extraction and curation (ST, CB, CC, MB, and EM), data analysis (ST and CB), manuscript preparation (ST), manuscript editing (ST, EM, and BP), and final manuscript approval (EM).

## Conflict of Interest Statement

The authors declare that the research was conducted in the absence of any commercial or financial relationships that could be construed as a potential conflict of interest.

## References

[1] Schulze-Bonhage A Epilepsy: The Intersection of Neurosciences, Biology, Mathematics, Engineering, and Physics. Boca Raton, FL: CRC Press (2011).

[2] Spencer SS Goncharova II Duckrow RB Novotny EJ Zaveri HP. Interictal spikes on intracranial recording: behavior, physiology, and implications. Epilepsia (2008) 49:1881–92. 10.1111/j.1528-1167.2008.01641.x 18479398

[3] Asano E Juhász C Shah A Sood S Chugani HT. Role of subdural electrocorticography in prediction of long-term seizure outcome in epilepsy surgery. Brain (2009) 132:1038–47. 10.1093/brain/awp025 19286694 PMC2668945

[4] Coutin-Churchman PE Wu JY Chen LL Shattuck K Dewar S Nuwer MR. Quantification and localization of EEG interictal spike activity in patients with surgically removed epileptogenic foci. Clin Neurophysiol (2012) 123:471–85. 10.1016/j.clinph.2011.08.007 21903463

[5] Greiner HM Horn PS Tenney JR Arya R Jain SV Holland KD Preresection intraoperative electrocorticography (ECoG) abnormalities predict seizure-onset zone and outcome in pediatric epilepsy surgery. Epilepsia (2016) 57:582–9. 10.1111/epi.13341 26918790

[6] Huang C Marsh ED Ziskind DM Celix JM Peltzer B Brown MW Leaving tissue associated with infrequent intracranial EEG seizure onsets is compatible with post-operative seizure freedom. J Pediatr Epilepsy (2012) 1:211–9. 10.3233/PEP-12033 24563805 PMC3930198

[7] Marsh ED Peltzer B Brown MW III Wusthoff C Storm PB Jr Litt B Interictal EEG spikes identify the region of electrographic seizure onset in some, but not all, pediatric epilepsy patients. Epilepsia (2010) 51:592–601. 10.1111/j.1528-1167.2009.02306.x 19780794 PMC2907216

[8] Barkmeier DT Loeb JA. An animal model to study the clinical significance of interictal spiking. Clin EEG Neurosci (2009) 40:234–8. 10.1177/155005940904000405 19780344 PMC2888497

[9] Chauvière L Doublet T Ghestem A Siyoucef SS Wendling F Huys R Changes in interictal spike features precede the onset of temporal lobe epilepsy. Ann Neurol (2012) 71:805–14. 10.1002/ana.23549 22718546

[10] Staley K Hellier JL Dudek FE. Do interictal spikes drive epileptogenesis? Neuroscientist (2005) 11:272–6. 10.1177/1073858405278239 16061513

[11] Staley KJ White A Dudek FE. Interictal spikes: harbingers or causes of epilepsy? Neurosci Lett (2011) 497:247–50. 10.1016/j.neulet.2011.03.070 21458535 PMC3124147

[12] Avoli M Do interictal discharges promote or control seizures? Experimental evidence from an in vitro model of epileptiform discharge. Epilepsia (2001) 42(Suppl 3):2–4. 10.1046/j.1528-1157.2001.042suppl.3002.x 11520313

[13] de Curtis M Avanzini G Interictal spikes in focal epileptogenesis. Prog Neurobiol (2001) 63:541–67. 10.1016/S0301-0082(00)00026-5 11164621

[14] Krishnan B Faith A Vlachos I Roth A Williams K Noe K Resetting of brain dynamics: epileptic versus psychogenic nonepileptic seizures. Epilepsy Behav (2011) 22(Suppl 1):S74–81. 10.1016/j.yebeh.2011.08.036 22078523 PMC3237405

[15] Badier JM Chauvel P. Spatio-temporal characteristics of paroxysmal interictal events in human temporal lobe epilepsy. J Physiol Paris (1995) 89:255–64. 10.1016/0928-4257(96)83642-4 8861824

[16] Baumgartner C Lindinger G Ebner A Aull S Serles W Olbrich A Propagation of interictal epileptic activity in temporal lobe epilepsy. Neurology (1995) 45:118–22. 10.1212/WNL.45.1.118 7824100

[17] Bourien J Bartolomei F Bellanger JJ Gavaret M Chauvel P Wendling F A method to identify reproducible subsets of co-activated structures during interictal spikes. Application to intracerebral EEG in temporal lobe epilepsy. Clin Neurophysiol (2005) 116:443–55. 10.1016/j.clinph.2004.08.010 15661121

[18] Wendling F Bartolomei F Senhadji L. Spatial analysis of intracerebral electroencephalographic signals in the time and frequency domain: identification of epileptogenic networks in partial epilepsy. Philos Trans A Math Phys Eng Sci (2009) 367:297–316. 10.1098/rsta.2008.0220 18957370 PMC2696099

[19] Hamandi K Powell HW Laufs H Symms MR Barker GJ Parker GJ Combined EEG-fMRI and tractography to visualise propagation of epileptic activity. J Neurol Neurosurg Psychiatry (2008) 79:594–7. 10.1136/jnnp.2007.125401 18096681 PMC2571962

[20] Kramer MA Cash SS. Epilepsy as a disorder of cortical network organization. Neuroscientist (2012) 18:360–72. 10.1177/1073858411422754 22235060 PMC3736575

[21] Lemieux L Daunizeau J Walker MC Concepts of connectivity and human epileptic activity. Front Syst Neurosci (2011) 5:12 10.3389/fnsys.2011.00012 21472027 PMC3065658

[22] Sabolek HR Swiercz WB Lillis KP Cash SS Huberfeld G Zhao G A candidate mechanism underlying the variance of interictal spike propagation. J Neurosci (2012) 32:3009–21. 10.1523/JNEUROSCI.5853-11.2012 22378874 PMC3319688

[23] Alarcon G Garcia Seoane JJ Binnie CD Martin Miguel MC Juler J Polkey CE Origin and propagation of interictal discharges in the acute electrocorticogram. Implications for pathophysiology and surgical treatment of temporal lobe epilepsy. Brain (1997) 120(Pt 12):2259–82. 10.1093/brain/120.12.2259 9448581

[24] Hufnagel A Dümpelmann M Zentner J Schijns O Elger CE. Clinical relevance of quantified intracranial interictal spike activity in presurgical evaluation of epilepsy. Epilepsia (2000) 41:467–78. 10.1111/j.1528-1157.2000.tb00191.x 10756415

[25] Lai Y van Drongelen W Hecox K Frim D Kohrman M He B. Cortical activation mapping of epileptiform activity derived from interictal ECoG spikes. Epilepsia (2007) 48:305–14. 10.1111/j.1528-1167.2006.00936.x 17295624

[26] Lee C Kim JS Jeong W Chung CK. Usefulness of interictal spike source localization in temporal lobe epilepsy: electrocorticographic study. Epilepsy Res (2014) 108:448–58. 10.1016/j.eplepsyres.2013.12.008 24434002

[27] Wilke C van Drongelen W Kohrman M He B. Identification of epileptogenic foci from causal analysis of ECoG interictal spike activity. Clin Neurophysiol (2009) 120:1449–56. 10.1016/j.clinph.2009.04.024 19616474 PMC2727575

[28] Wieser HG Blume WT Fish D Goldensohn E Hufnagel A King D ILAE Commission Report. Proposal for a new classification of outcome with respect to epileptic seizures following epilepsy surgery. Epilepsia (2001) 42:282–6. 10.1046/j.1528-1157.2001.4220282.x 11240604

[29] Martinet LE Ahmed OJ Lepage KQ Cash SS Kramer MA. Slow spatial recruitment of neocortex during secondarily generalized seizures and its relation to surgical outcome. J Neurosci (2015) 35:9477–90. 10.1523/JNEUROSCI.0049-15.2015 26109670 PMC4478258

[30] Brown MW III Porter BE Dlugos DJ Keating J Gardner AB Storm PB Jr Comparison of novel computer detectors and human performance for spike detection in intracranial EEG. Clin Neurophysiol (2007) 118:1744–52. 10.1016/j.clinph.2007.04.017 17544322

[31] Lorenz MO Methods of measuring the concentration of wealth. Publ Am Stat Assoc (1905) 9:209–19. 10.2307/2276207

[32] Zumsteg D Friedman A Wieser HG Wennberg RA. Propagation of interictal discharges in temporal lobe epilepsy: correlation of spatiotemporal mapping with intracranial foramen ovale electrode recordings. Clin Neurophysiol (2006) 117:2615–26. 10.1016/j.clinph.2006.07.319 17029950

[33] Hung C-C Peng W-C Lee W-C Clustering and aggregating clues of trajectories for mining trajectory patterns and routes. VLDB J (2011) 24:169–92. 10.1007/s00778-011-0262-6

[34] Bullmore E Sporns O. Complex brain networks: graph theoretical analysis of structural and functional systems. Nat Rev Neurosci (2009) 10:186–98. 10.1038/nrn2575 19190637

[35] Moran PA Notes on continuous stochastic phenomena. Biometrika (1950) 37:17–23. 10.2307/2332142 15420245

[36] Asano E Juhász C Shah A Muzik O Chugani DC Shah J Origin and propagation of epileptic spasms delineated on electrocorticography. Epilepsia (2005) 46:1086–97. 10.1111/j.1528-1167.2005.05205.x 16026561 PMC1360692

[37] Malinowska U Badier JM Gavaret M Bartolomei F Chauvel P Bénar CG. Interictal networks in magnetoencephalography. Hum Brain Mapp (2014) 35:2789–805. 10.1002/hbm.22367 24105895 PMC6869550

[38] Ossadtchi A Mosher JC Sutherling WW Greenblatt RE Leahy RM. Hidden Markov modelling of spike propagation from interictal MEG data. Phys Med Biol (2005) 50:3447–69. 10.1088/0031-9155/50/14/017 16177520

[39] Tanaka N Stufflebeam SM. Clinical application of spatiotemporal distributed source analysis in presurgical evaluation of epilepsy. Front Hum Neurosci (2014) 8:62. 10.3389/fnhum.2014.00062 24574999 PMC3919017

[40] van Mierlo P Papadopoulou M Carrette E Boon P Vandenberghe S Vonck K Functional brain connectivity from EEG in epilepsy: seizure prediction and epileptogenic focus localization. Prog Neurobiol (2014) 121:19–35. 10.1016/j.pneurobio.2014.06.004 25014528

[41] Yaffe RB Borger P Megevand P Groppe DM Kramer MA Chu CJ Physiology of functional and effective networks in epilepsy. Clin Neurophysiol (2015) 126:227–36. 10.1016/j.clinph.2014.09.009 25283711

[42] Panzica F Varotto G Rotondi F Spreafico R Franceschetti S. Identification of the epileptogenic zone from stereo-EEG signals: a connectivity-graph theory approach. Front Neurol (2013) 4:175. 10.3389/fneur.2013.00175 24223569 PMC3818576

[43] van Mierlo P Carrette E Hallez H Raedt R Meurs A Vandenberghe S Ictal-onset localization through connectivity analysis of intracranial EEG signals in patients with refractory epilepsy. Epilepsia (2013) 54:1409–18. 10.1111/epi.12206 23647147

[44] Khambhati AN Davis KA Oommen BS Chen SH Lucas TH Litt B Dynamic network drivers of seizure generation, propagation and termination in human neocortical epilepsy. PLoS Comput Biol (2015) 11(12):e1004608 10.1371/journal.pcbi.1004608 26680762 PMC4682976

[45] Antony AR Alexopoulos AV González-Martínez JA Mosher JC Jehi L Burgess RC Functional connectivity estimated from intracranial EEG predicts surgical outcome in intractable temporal lobe epilepsy. PLoS One (2013) 8:e77916. 10.1371/journal.pone.0077916 24205027 PMC3813548

