# Supplementary material for: Spatiotemporal Mapping of Interictal Spike Propagation: A Novel Methodology Applied to Pediatric Intracranial EEG Recordings
Source: Front Neurol. 2016 Dec 19;7:229. doi: 10.3389/fneur.2016.00229 (PMC5165024; doi:10.3389/fneur.2016.00229)
Supplement: Supplementary file 1 [file data_sheet_1.pdf]

**frontiers**

# Spatiotemporal mapping of interictal spike propagation: a novel methodology applied to pediatric intracranial EEG recordings

**Samuel B. Tomlinson, Camilo Bermudez, Chiara Conley, Merritt W. Brown, Brenda E. Porter, Eric D. Marsh***

* **Correspondence:** Eric D. Marsh, MD PhD: marshe@email.chop.edu

## 1. Supplementary Figure

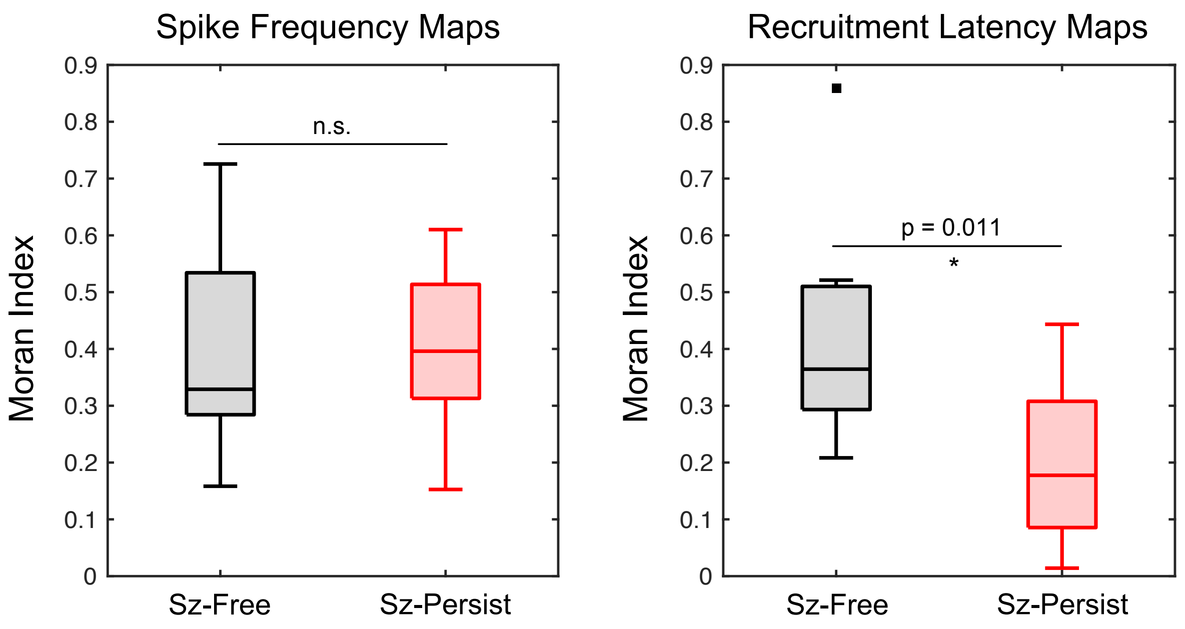


**Supplementary Figure 1. Results from the spike frequency analysis and the recruitment latency analysis were preserved when 50,000 spikes / patient were assessed.** In the main analysis, two patients (Pt 01, Pt 08) had only 50,000 interictal spikes available. This figure illustrates that the key patterns of results observed in the main analysis are preserved when 50,000 spikes (rather than 100,000 spikes) were analyzed for all patients.
